# Rise of the alt‐White? Examining the prevalence of perceived racial and gender discrimination among White men from 2014 to 2023

**DOI:** 10.1111/bjso.70010

**Published:** 2025-08-28

**Authors:** Kieren J. Lilly, Chantelle Kimberley, Zoe Bertenshaw, Joaquín Bahamondes, Chris G. Sibley, Danny Osborne

**Affiliations:** ^1^ Institute for Social Science Research University of Queensland Brisbane Queensland Australia; ^2^ School of Psychology University of Auckland Auckland New Zealand; ^3^ Escuela de Psicología Universidad Católica del Norte Antofagasta Chile

**Keywords:** ethnicity, ideology, latent class growth analysis, perceived discrimination, status threat

## Abstract

The alt‐right increasingly claims that White men are becoming targets of discrimination, yet few studies examine how, and for whom, perceived (reverse) discrimination manifests among White men. We address this oversight by examining rates of change in perceptions of ethnic and gender discrimination across 10 annual waves of a nationwide sample of White men (2014 to 2023; *N* = 20,486). Latent class growth analysis revealed that most White men (82.75% of participants) reported low and stable perceptions of discrimination over time, alleviating concerns of widespread discontent. However, we identified a Disenfranchised class (8.49%) that perceived moderate discrimination and a Radicalized class (8.76%) whose initially low levels of perceived discrimination increased markedly over time. These classes differed across socio‐demographic variables, socio‐political attitudes and well‐being measures. We thus identify how, and for whom, perceptions of discrimination change over time among White men and how these changes undermine health and progressive social change.

## INTRODUCTION

Social movements such as Black Lives Matter and #MeToo have catalysed societal discussions of racial and gender‐based discrimination (see Hillstrom, 2018; Szetela, 2020). However, these discussions have sparked resistance from movements advocating for the rights of structurally *advantaged* groups (Becker, 2020; Mondon & Winter, 2020). Indeed, resistance to social progress is marked by claims that advances for minority groups threaten the rights of dominant groups (see Boehme & Isom Scott, 2020; Norton & Sommers, 2011). Concerns of ‘anti‐White’ and ‘anti‐male’ biases are particularly at the forefront of right‐wing political campaigns (Hughey, 2014; Rhodes, 2010) and movements (see Simi et al., 2024). Critically, political figures such as U.S. President Donald Trump capitalize on concerns of anti‐White discrimination to attract support, especially from White men (for discussion, see Inwood, 2019; Turney et al., 2017). As such, perceptions of (reverse) discrimination among advantaged groups are of increasing concern in contemporary politics.

Despite increasing *claims* of reverse discrimination in public discourse, few studies directly examine whether advantaged group members feel discriminated against, nor whether these perceptions are changing over time. Most empirical studies of perceived discrimination among advantaged groups are cross‐sectional (e.g. Isom Scott, 2018; Nelson et al., 2018; Okuyan et al., 2023; although note Okuyan and colleagues utilized data from two timepoints). The few studies that *do* investigate perceived discrimination longitudinally focus on its relationships with ideology (e.g. Bahamondes et al., 2021a) or health outcomes (for a meta‐analytic review, see Schmitt et al., 2014) rather than how perceptions of discrimination develop and change across time. Examining these processes is necessary to increase understanding of how perceived reverse discrimination manifests in the population. Moreover, the narrative that advantaged groups are ‘victims’ of discrimination is often used to deflect responsibility for harm towards disadvantaged groups (Noor et al., 2012). Accordingly, understanding the extent to which advantaged groups feel discriminated against—and whether this perception is increasing over time—is essential for monitoring intergroup conflict and determining how White (male) privilege is sustained.

Critically, we must consider *who* among advantaged groups perceives reverse discrimination. Indeed, many advantaged group members acknowledge their objective privileges and express solidarity with *dis*advantaged groups (under certain conditions; see Halvorsen et al., 2025; Radke et al., 2020). For instance, many White Americans and men fight for racial justice (Russo, 2023) and gender equality (Messner et al., 2015), respectively. In New Zealand (the country of the present study), most New Zealand Europeans are unsupportive of European‐focused collective action (Lilly et al., 2025) and many engage in anti‐racism activism (e.g. Hancock & Newton, 2022; Showden et al., 2022). These results indicate that perceptions of reverse discrimination can vary widely within advantaged groups.

Differences may also emerge among those who *do* feel discriminated against. For example, some White men may perceive reverse discrimination because they are threatened by social progress for minority groups (Cohrs & Asbrock, 2009; Schmitt & Branscombe, 2002; Wilkins & Kaiser, 2014), whereas others may (inaccurately) use reverse discrimination narratives to explain other forms of disadvantage (such as social class; Roediger, 1991; Turney et al., 2017). Identifying the distinct ‘types’ of people most susceptible to the rhetoric of reverse discrimination would further elucidate the processes underlying perceived discrimination and help develop targeted interventions that reduce these perceptions among advantaged groups. In short, nuanced understandings of perceived reverse discrimination among advantaged groups require approaches that can identify (a) the prevalence of perceived reverse discrimination (or lack thereof) within advantaged groups and (b) *who* among advantaged groups is most likely to perceive reverse discrimination.

### The current study

The current study meets this need by presenting an exploratory analysis of perceived ethnic and gender discrimination across 10 annual waves of a nationwide random sample of New Zealand European (hereafter White) men. We focus on White men because they are the primary beneficiaries of White supremacy and the intended targets of right‐wing movements that employ reverse discrimination or victim narratives, particularly in Western democracies (Boehme & Isom Scott, 2020; Turney et al., 2017). Moreover, status threat among White women differs from men via its roots in both oppression and privilege (see Isom Scott et al., 2022, for discussion). We thus narrow our focus to White men as an advantaged ethnic *and* gender group.

New Zealand provides an ideal context to examine our research questions given the pervasive inequities between majority and minority ethnic groups (particularly between New Zealand Europeans and Māori, the Indigenous peoples; see Ministry of Social Development, 2016), and genders (e.g. Stephens et al., 2022). Despite New Zealand's egalitarian reputation, these inequities—and the colonial biases of New Zealand's social structures (Bell, 2006)—keep debates of White (male) privilege at the forefront of mainstream discourse. In short, our focus on White men in New Zealand allows us to examine a group whose denial of privilege ultimately contributes to the maintenance of colonial inequalities. Below, we provide an overview of our focal research questions and predictions.

#### Research question 1: How are perceptions of reverse discrimination changing among White men?

We first examine how perceived discrimination is changing over time among White men. To do so, we use latent class growth analysis (LCGA), a person‐centred longitudinal method that allows researchers to determine whether different subgroups of a sample differ in their levels of, and changes in, a construct over time. In the present study, this approach tests the possibility that only *some* White men are increasing in their perceptions of discrimination over time, whereas others may report low or stable perceptions. Notably, the time period of our study (2014 to 2023) allows us to examine trends in perceived discrimination following events such as Trump's inaugural presidential election (2016), the international proliferation of #MeToo (2017) and Black Lives Matter (2020), the Christchurch terror attack (2019) and the COVID‐19 pandemic (2020). In doing so, we examine perceptions of discrimination in a time of increased tensions between structurally advantaged and disadvantaged groups and rising right‐wing rhetoric (Simi et al., 2024). Although our analyses are exploratory, these increased tensions suggest we will identify at least one subgroup whose perceptions of reverse discrimination increase over time.

#### Research question 2: Who is most likely to experience changes in perceived reverse discrimination?

In addition to exploring different trends in perceived discrimination among White men, we examine the demographic and socio‐political correlates of any distinct subgroups. Specifically, we consider potential (a) socio‐demographic, (b) identity centrality and (c) ideological differences across classes.

##### Socio‐demographic factors

Prior research suggests that White people who feel ‘left‐behind’ socioeconomically may explain their disadvantage via racial discourses. For example, Okuyan et al. (2023) found that objective income and *perceived* financial insecurity predict perceived discrimination among White Americans. However, research examining the links between perceived relative deprivation and prejudice suggests that both the relatively poor *and* relatively wealthy express comparable attitudes (Anier et al., 2016), and that fear of *future* disadvantage explains this phenomenon among the wealthy (e.g. Jetten et al., 2015). Thus, socioeconomically disadvantaged and advantaged group members may perceive similar levels of reverse discrimination—albeit for different reasons (see Simi et al., 2024; Turney et al., 2017). To test this possibility, we explore whether perceived relative deprivation, home ownership (for discussion, see Foye et al., 2018), and education correlate with distinct trajectories of perceived reverse discrimination. Additionally, we examine the role of migrant status, as prior research suggests that migrant status affects perceptions of group‐based disadvantage (although evidence is mixed; Jin, 2016; Obaidi et al., 2019). Finally, we examine the effects of age and sexual minority status, as recent research suggests that young heterosexual men may be more susceptible to alt‐right narratives than their older and sexual minority counterparts (Botto & Gottzén, 2024).

##### Identity centrality

Research suggests that the strength (identification) and importance (centrality) of one's group identity influences their sensitivity to status threats and perceived discrimination. Indeed, group identification predicts perceived group injustice (Thomas et al., 2012; Thomas et al., 2020; Zubielevitch et al., 2020), and identity *centrality* predicts perceived gender discrimination among men (Cameron, 2001). However, evidence for the relationship between group identity and perceived ethnic discrimination among White people remains mixed (Major et al., 2002; Okuyan et al., 2023). Similar to socioeconomic factors, these inconsistencies may be due to different subsets of an advantaged group perceiving discrimination for distinct reasons. We thus explore class‐based differences in gender and ethnic identity centrality.

##### Ideological factors

Finally, we explore class differences in ideology and conspiracy mentality. Prior research suggests that advantaged group members who endorse traditional social systems and group‐based hierarchies (Bahamondes et al., 2021b) and race‐based conspiracy beliefs (Jolley et al., 2020; Obaidi et al., 2022) are more likely to perceive discrimination and express prejudice towards minority groups. For instance, Bahamondes et al. (2021b) found that social dominance orientation predicts perceived discrimination among New Zealand Europeans (see also Okuyan & Vollhardt, 2022), whereas Okuyan et al. (2023) demonstrate that system‐legitimizing beliefs (such as conservatism and symbolic threat) positively correlate with perceived discrimination among White Americans. That said, recent research suggests that advantaged groups *low* in system‐justifying beliefs express greater support for reactionary movements (e.g. movements concerned with the rights of structurally advantaged groups), likely because they perceive existing social systems as ‘failing’ to protect their interests (Liekefett & Becker, 2022; Lilly et al., 2025). Similarly, research suggests people's endorsement of conspiracy beliefs, perceptions of legitimacy and support for reactionary movements are related (Thomas et al., 2024). In sum, these ideologies may have differential associations with perceived discrimination among different subgroups. We explore this possibility by testing for class differences in right‐wing authoritarianism, social dominance orientation, conservatism, system justification and conspiracy mentality.

#### Research question 3: How do changes in perceived reverse discrimination correlate with socio‐political attitudes and well‐being?

Our final analysis examines differences across classes in their mean attitudes towards disadvantaged groups (namely, women, sexual minorities, migrants, ethnic minorities and Indigenous peoples), support for progressive policies, political party preferences and well‐being. If we identify distinct groups that differ in their trajectories of perceived discrimination over time, these groups should also differ across these core variables. Indeed, perceived disadvantage motivates group members to improve their group's status (e.g. Wright et al., 1990). Among advantaged groups, this manifests as the devaluation of disadvantaged groups that ostensibly threaten their societal privileges (Gheorghiu et al., 2022; Taylor, 2002). Thus, White men who perceive their group as disadvantaged should report more negative attitudes towards minority groups. These subgroups of White men should also express greater support for right‐wing political parties because these parties attract support with campaigns to ‘fight’ reverse discrimination and diversity initiatives (Inwood, 2019; Major et al., 2018)—including in New Zealand (e.g. Craymer, 2023). Finally, because perceived discrimination predicts ill health (see Schmitt et al., 2014), White men who perceive reverse discrimination may also report lower well‐being. We test this possibility via measures of psychological distress, meaning in life, felt belongingness, satisfaction with future security and subjective health. Collectively, the present study provides novel insights into (a) how perceptions of reverse discrimination may be changing among White men, (b) for whom these changes are most salient and (c) the potential consequences these differing trajectories of perceived discrimination have on socio‐political attitudes and well‐being.

## METHOD

### Data and materials

We use data from the New Zealand Attitudes and Values Study (NZAVS), a nationwide longitudinal panel study of New Zealand adults. The NZAVS was approved by the University of Auckland Human Participants Ethics Committee, and informed consent was obtained from participants. Full copies of the NZAVS data files are held by all members of the NZAVS management team and advisory board. A de‐identified dataset containing the variables analysed in this manuscript is available upon request from the corresponding author, or any member of the NZAVS advisory board for replication or checking of any published study using NZAVS data. Information about the measures, sampling procedure, retention rates and ethics approvals for the NZAVS, as well as Mplus syntax for all models reported in this study, is available via the Open Science Framework: https://osf.io/75snb/.

### Sampling procedure and participants

NZAVS participants were initially randomly sampled from the electoral roll (Time 1, 2009 *N* = 6518, response rate: 16.6%). To diversify and increase the size of the sample, booster sampling was conducted at Time 3 (2011; *N* = 6884, *n*_booster_ = 2966), Time 4 (2012; *N* = 12,179, *n*_booster_ = 5371), Time 5 (2013; *N* = 18,261, *n*_booster_ = 7757), Time 8 (2016; *N* = 21,936, *n*_booster_ = 8270), Time 10 (2018; *N* = 47,948, *n*_booster_ = 29,291), Time 11 (2019; *N* = 42,681, *n*_booster_ = 6154), Time 14 (2022; *N* = 33,722, *n*_booster_ = 2041) and Time 15 (2023; *N* = 32,857, *n*_booster_ = 3293). By Time 15 (2023), 76,409 participants had completed at least one wave of the study, with good wave‐to‐wave retention (67.9–85.6%).

Although the NZAVS began in 2009, we first assessed perceived gender‐based discrimination at Time 6 (2014). As such, we focus on the 20,486^1^ sole‐identifying White men who completed one or more waves of the NZAVS from Time 6 (2014) to Time 15 (2023; *M*_waves_ = 4.28, *SD* = 2.66; see Table 1 for further information). Most of these participants were born in New Zealand (78.3%), and the average age at Time 6 (2014) was 45.83 (*SD* = 15.92). Table 1 displays a further breakdown of sample characteristics at each measurement occasion.

**TABLE 1:** Sample demographics across assessments.

Variable	Time 6	Time 7	Time 8	Time 9	Time 10	Time 11	Time 12	Time 13	Time 14	Time 15
Sample size, *n*	4518	4068	6433	5005	14,206	12,486	11,370	10,040	10,017	9503
Sample size (first response)^a^	4518	349	2584	82	8953	1696	100	416	1018	770
% of full sample (*N* = 20,486)	(22.1%)	(1.7%)	(12.6%)	(0.4%)	(43.7%)	(8.3%)	(0.5%)	(2.0%)	(5.0%)	(3.8%)
Sample size (last response)^a^	295	233	561	365	2081	1400	1424	1608	3016	9503
% of full sample (*N* = 20,486)	(1.4%)	(1.1%)	(2.7%)	(1.8%)	(10.2%)	(6.8%)	(7.0%)	(7.8%)	(14.7%)	(46.4%)
Age	52.47	53.92	52.14	54.08	50.83	53.81	55.39	56.97	56.28	55.93
*SD*	(14.19)	(13.96)	(13.97)	(13.63)	(13.70)	(13.50)	(13.23)	(13.10)	(15.22)	(16.32)
Born in New Zealand (yes)	78.6%	79.1%	78.8%	78.6%	79.1%	77.7%	77.8%	77.4%	79.1%	78.0%
Household income^b^	1.16	1.19	1.19	1.25	1.27	1.30	1.32	1.37	1.40	1.40
*SD*	(1.03)	(0.97)	(1.02)	(1.03)	(1.06)	(1.28)	(1.21)	(1.35)	(1.56)	(1.44)
Homeowner (yes)	–	82.1%	–	–	76.9%	–	82.1%	–	79.8%	77.6%
Sexual orientation										
Heterosexual	79.9%	84.2%	85.8%	91.6%	90.0%	91.7%	91.7%	92.7%	90.8%	90.2%
Gay	2.3%	2.5%	3.0%	3.1%	3.0%	3.7%	3.8%	4.0%	4.0%	4.2%
Bisexual	1.1%	1.5%	1.6%	1.7%	1.8%	2.1%	2.3%	2.2%	2.7%	3.2%
Bicurious	0.5%	0.6%	0.6%	0.4%	0.3%	0.4%	0.3%	0.4%	0.4%	0.4%
Pansexual	0.2%	0.3%	0.4%	0.3%	0.2%	0.3%	0.3%	0.3%	0.5%	0.5%
Asexual	0.1%	0.2%	0.2%	0.1%	0.1%	0.2%	0.3%	0.2%	0.3%	0.4%
Missing/Outside scope	15.9%	10.7%	8.4%	2.8%	4.6%	1.6%	1.3%	0.2%	1.3%	1.1%
Perceived ethnic discrimination^c^	1.73	1.73	1.73	1.76	1.81	1.87	2.04	2.02	2.06	1.97
*SD*	(1.22)	(1.22)	(1.25)	(1.26)	(1.36)	(1.41)	(1.49)	(1.59)	(1.63)	(1.53)
Perceived gender discrimination^c^	1.74	1.76	1.76	1.86	1.87	1.94	2.22	1.94	1.99	1.94
*SD*	(1.19)	(1.22)	(1.23)	(1.33)	(1.35)	(1.41)	(1.56)	(1.44)	(1.50)	(1.46)

^a^ Total participants who first and last completed the NZAVS survey at the given time point.

^b^ Household income divided by NZD$100,000.

^c^ Measured on a 1 (strongly disagree) to 7 (strongly agree) scale.

### Measures

Our focal measures were embedded within the larger NZAVS questionnaire and were based on short‐form scales due to space constraints. However, short‐form scales used in the NZAVS survey have been validated against their full‐form parent counterparts (where applicable), with all measures displaying at least adequate reliability and factor loadings (Sibley et al., 2024). Unless otherwise specified, participants rated their agreement with items on a 1 (*strongly disagree*) to 7 (*strongly agree*) scale at every assessment occasion (Time 6 to Time 15).

#### Perceived discrimination

Perceived ethnic discrimination was measured using the item: ‘I feel that I am often discriminated against because of my ethnicity’. Perceived gender discrimination was measured using the item: ‘I feel that I am often discriminated against because of my gender’.

#### Predictors

To facilitate comparisons across predictors and place our measures on a common metric, all variables were recoded to a 0 to 1 scale.

##### Demographic covariates

Participants reported their age, education, sexual orientation and whether they were born in New Zealand (0 = no, 1 = yes) and owned their own home. Education was measured by using participants' highest level of qualification, with responses coded into an 11‐point ordinal variable according to the New Zealand Qualifications Authority (0 = no formal qualification, 10 = doctoral degree or equivalent). Sexual orientation was assessed with the question, ‘How would you describe your sexual orientation?’, with responses dummy‐coded based on whether participants had ever reported a sexual minority identity (0 = heterosexual, 1 = sexual minority). Home ownership was assessed at Time 7, Time 10, Time 12, Time 14 and Time 15 using the forced choice yes/no item: ‘Do you own your own home? (either partially or fully owned)’. We dummy‐coded participants' responses based on whether they had ever reported owning their own home (0 = no, 1 = yes).

To maximize the number of unique cases for predictors assessed at multiple waves, we assessed the following measures using responses from each participant's *first* assessment occasion. For instance, we used data from Time 7 for participants who first entered the study at Time 7.

**Individual‐based relative deprivation** was measured using the mean of two items adapted from Abrams and Grant (2012): (a) ‘I'm frustrated by what I earn relative to other people in New Zealand’ and (b) ‘I generally earn less than other people in New Zealand’ (Spearman's rho (*r*_s_) = .42–.47, *p*s < .001).

**Group‐based relative deprivation** was measured using the mean of two items adapted from Abrams and Grant (2012): (a) ‘I'm frustrated with what my ethnic group earns relative to other groups in New Zealand’ and (b) ‘People from my ethnic group generally earn less than other groups in New Zealand’ (*r*_s_ = .30–.40, *p*s < .001).

**Ethnic identity centrality** was measured using the mean of three items from Leach et al. (2008): (a) ‘I often think about the fact that I am a member of my ethnic group’; (b) ‘The fact that I am a member of my ethnic group is an important part of my identity’; and (c) ‘Being a member of my ethnic group is an important part of how I see myself’ (*ω*s = .76–.82).

**Gender identity centrality** was measured from Times 10 through 15 using a single item adapted from Leach et al. (2008): ‘Being a woman/man is an important part of how I see myself’.

**Conservatism** was measured by asking participants: ‘Please rate how politically liberal versus conservative you see yourself as being’ on a 1 (*extremely liberal*) to 7 (*extremely conservative*) scale.

**System justification** was measured using the mean of four items adapted from Kay and Jost (2003): (a) ‘In general, I find New Zealand society to be fair’; (b) ‘In general, the New Zealand political system operates as it should’; (c) ‘Everyone has a fair shot of wealth and happiness in New Zealand’; and (d) ‘Most of New Zealand's policies serve the greater good’ (*ω*s = .69–.79).

**Social dominance orientation** was assessed using the mean of six items from Sidanius and Pratto's (2001) 16‐item SDO_6_ scale: (a) ‘It is OK if some groups have more of a chance in life than others’; (b) ‘Inferior groups should stay in their place’; (c) ‘To get ahead in life, it is sometimes okay to step on other groups’; (d) ‘We should have increased social equality’ (reverse‐scored); (e) ‘It would be good if groups could be equal’ (reverse‐scored); and (f) ‘We should do what we can to equalize conditions for different groups’ (reverse‐scored; *ω*s = .72–.79).

**Right‐wing authoritarianism** was measured using the mean of six items from Altemeyer's (1996) 30‐item scale: (a) ‘It is always better to trust the judgment of the proper authorities in government and religion than to listen to the noisy rabble‐rousers in our society who are trying to create doubt in people's minds’; (b) ‘It would be best for everyone if the proper authorities censored magazines so that people could not get their hands on trashy and disgusting material’; (c) ‘Our country will be destroyed some day if we do not smash the perversions eating away at our moral fibre and traditional beliefs’; (d) ‘People should pay less attention to The Bible and other old traditional forms of religious guidance, and instead develop their own personal standards of what is moral and immoral’ (reverse‐scored); (e) ‘Atheists and others who have rebelled against established religions are no doubt every bit as good and virtuous as those who attend church regularly’ (reverse‐scored); and (f) ‘Some of the best people in our country are those who are challenging our government, criticizing religion and ignoring the ‘normal way’ things are supposed to be done’ (reverse‐scored; *ω*s = .65–.77).

**Conspiracy belief mentality** was measured from Times 11 through 15 using a single item from Lantian et al. (2016): ‘I think that the official version of major world events given by authorities often hides the truth’.

#### Socio‐political and well‐being measures

To maximize the unique cases for our outcome variables, we assessed the following measures using responses from each participant's *most recent* assessment occasion. All variables were recoded to a 0 to 1 scale to facilitate comparisons across outcomes.

**Benevolent sexism** was assessed using the mean of five items from Glick and Fiske's (1996) Ambivalent Sexism Inventory: (a) ‘Women, compared to men, tend to have greater moral sensibility’; (b) ‘Many women have a quality of purity that few men possess’; (c) ‘Women, as compared to men, tend to have a more refined sense of culture and good taste’; (d) ‘Women should be cherished and protected by men’; and (e) ‘Every man ought to have a woman whom he adores’ (*ω*s = .67–.73).

**Hostile sexism** was assessed using the mean of five items from Glick and Fiske's (1996) Ambivalent Sexism Inventory: (a) ‘Women are too easily offended’; (b) ‘Women exaggerate problems they have at work’; (c) ‘Women seek to gain power by getting control over men’; (d) ‘Once a woman gets a man to commit to her she usually tries to put him on a tight leash’; and (e) ‘When women lose to men in a fair competition, they typically complain about being discriminated against’ (*ω*s = .82–.87).

**Sexual prejudice** was assessed using one item: ‘I think that homosexuality should be accepted by society’ (reverse‐scored).

**Support for sexual violence education** was assessed from Time 8 through Time 11, using two items developed for the NZAVS to assess support for preventing men's violence towards women by educating men and women, respectively. Sexual violence education targeting men was assessed using the item: ‘We should invest more in educating men to not be physically/sexually violent towards women’. Sexual violence education targeting women was assessed using the item: ‘We should invest more in educating women how to avoid physical/sexual violence from men’ (Brownhalls et al., 2021).

**Abortion attitudes** were assessed using two items adapted from the General Social Survey (Smith et al., 2020). One item measured support for elective abortion: ‘Legalized abortion for women, regardless of the reason’. One item measured support for traumatic abortion: ‘Legalized abortion when the woman's life is endangered’.

**Multi‐culturalism attitudes** were assessed using the mean of three items: (a) ‘The unity of New Zealand is weakened by too many immigrants’ (reverse‐scored); (b) ‘I feel at ease when I am in a city district in New Zealand with many immigrants’; and (c) ‘There are too many immigrants living in New Zealand’ (reverse‐scored; *ω*s = .78–.83).

**Support for Māori political mobilization** was assessed using the mean support for two items: (a) ‘Protest marches and public demonstrations supporting the rights of Māori’; and (b) ‘Māori have too much political power and influence in decisions affecting New Zealand’ (reverse‐scored; *r*_s_ = .60–.71, *p*s < .001).

**Modern racism towards Māori** was assessed using a single item: ‘Discrimination against Māori is no longer a problem in New Zealand’.

**Collective action support on behalf of Europeans** was assessed using the mean of three items from Cronin et al. (2012): (a) ‘I have considered voting in terms of what is good for my particular ethnic group’; (b) ‘I have considered participating in demonstrations on behalf of my ethnic group’; and (c) ‘I have considered signing petitions on behalf of my ethnic group’ (*ω*s = .67–.75).

**Feeling thermometer ratings** (i.e. warmth) of New Zealand Europeans, Māori, Pacific Islanders, immigrants, Asians (in general), Chinese people, Indians, Muslims and refugees^2^ were assessed using a scale modelled on the American National Election Study. We asked participants to ‘Please rate your feelings of warmth towards the following groups using the ‘feeling thermometer scale’” for each group on a 1 (*least warm*) to 7 (*most warm*) scale.

**Political party support** was assessed by asking participants to ‘rate how strongly you oppose or support each of the following parties’ on a 1 (*strongly oppose*) to 7 (*strongly support*) scale. The parties included National (New Zealand's centre‐right party), Labour (New Zealand's centre‐left party), ACT (Association of Consumers and Taxpayers; a right‐wing classical‐liberal party), Green (a left‐wing party), Te Pāti Māori (the Māori party; a left‐wing progressive party) and New Zealand First (a small nationalistic right‐wing populist party).

**Psychological distress** was assessed using Kessler et al.'s (2010) K6 psychological distress scale. The scale contains six items measured on a 0 (*none of the time*) to 4 (*all of the time*) scale, with participants reporting how often they felt the following in the last 30 days: (a) ‘hopeless?’; (b) ‘so depressed that nothing could cheer you up?’; (c) ‘restless or fidgety?’; (d) ‘that everything was an effort?’; (e) ‘worthless?’; and (f) ‘nervous?’ (*ω*s = .83–.86).

**Meaning in life** was assessed from Times 10 through 15 using the mean of two items from Steger et al. (2006): (a) ‘My life has a clear sense of purpose’ and (b) ‘I have a good sense of what makes my life meaningful’ (*r*_s_ = .59–.64, *p*s < .001).

**Felt belongingness** was assessed using the mean of three items adapted from Hagerty and Patusky (1995). Participants were asked to rate how accurately the following statements described them: I… (a) ‘…Know that people in my life accept and value me’; (b) ‘…Feel like an outsider’ (reverse‐scored); and (c) ‘…Know that people around me share my attitudes and beliefs’ (*ω*s = .57–.62).

**Satisfaction with future security** was assessed by asking participants to rate their satisfaction with their ‘future security’ on a 0 (*completely dissatisfied*) to 10 (*completely satisfied*) scale.

**Subjective health** was assessed using the mean of two items from Ware and Sherbourne (1992). Participants were asked (a) ‘In general, would you say your health is…’ and asked to rate their health on a scale from 1 (*poor*) to 7 (*excellent*). Participants were also asked to rate their agreement with (b) ‘I seem to get sick a little easier than other people’ (reverse‐scored; *r*_s_ = .36–.40, *p*s < .001).

### Analytic approach

The present study examined the prevalence of, and rates of change in, perceived discrimination among White men over 9 years (from 2014 to 2023). To do so, we used latent class growth analyses (LCGA) in Mplus *v*.8.11 (Muthén & Muthén, 1998–2024) with full‐information maximum likelihood estimation (FIML) to account for missing data (Enders & Bandalos, 2001). LCGA allows for the possibility that different subsets of the population differ in their rates of change in a construct over time (see Muthén & Muthén, 2000) and, accordingly, allows us to test the possibility that different subgroups of White men differ in their mean levels *and* trajectories of perceived discrimination over time. In the present study, we estimate multi‐trajectory classes based on the trajectories of perceived ethnic *and* gender discrimination to elucidate potential combinations of these two constructs.

**FIGURE 1:**
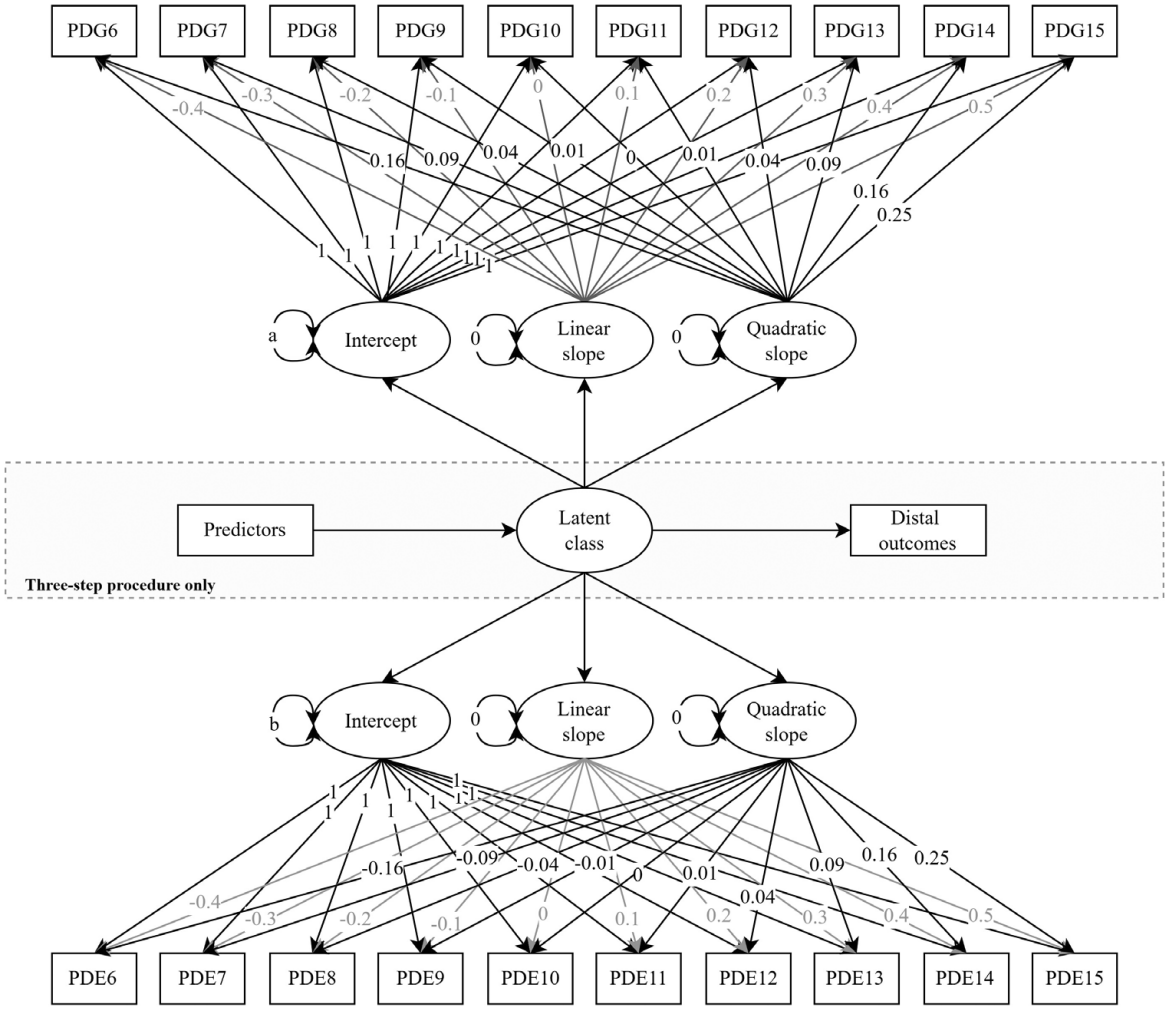
Structural equation modelling representation of a multi‐trajectory latent class growth model of perceived ethnic and gender discrimination. The figure depicts a multi‐trajectory latent class growth model estimating latent intercepts, linear slopes and quadratic slopes for perceived ethnic (PDE) and gender (PDG) discrimination across ten annual assessments. The model is centred at Time 10, and variances of the intercepts are constrained to equality across classes (denoted by ‘a’ and ‘b’ for PDG and PDE, respectively). For display purposes, residual variances are omitted from the figure, although they were constrained across assessments and classes in the model. Linear slope paths are denoted in grey for readability. Paths within the dashed box depict the three‐step procedures for predictors (left‐hand side) and distal outcomes (right‐hand side).

We first estimated a model with a one‐class trajectory, which assumes the entire sample falls within a single homogenous group (see Figure 1 for a graphical representation). We then estimated models fitting between 2 and 6 classes. For each model, we estimated intercepts and rates of change for perceived ethnic and gender discrimination in each class. We estimated both linear and quadratic rates of change for each class to account for non‐linear changes in our focal constructs over time. We then (a) freely estimated the variances of the intercepts but constrained these to equality across classes and (b) constrained to equality the residual variances across classes and assessment occasions. Because LCGA assumes that individuals within each identified class are homogenous in their trajectories over time (Muthén, 2004; Muthén & Muthén, 2000), we also constrained the growth variances and covariances to 0.

To determine the best‐fitting model for our data, we considered the theoretical contributions of any identified classes (including their size and distinct features relative to other classes) alongside formal model fit criteria. These fit criteria included the Akaike Information Criterion (AIC), the Bayesian Information Criterion (BIC) and the sample size adjusted BIC (aBIC), where lower values indicate relatively better model fit (Muthén & Muthén, 2000). We also inspected each model's classification precision (i.e. entropy), with values closer to 1.0 indicating a more precise separation of our data into distinct classes (Collins & Lanza, 2010). Finally, we validated our solutions by replicating models in two randomly split halves of the sample; if the identified classes reflect meaningful subgroups in the population, they should replicate in random subsets of our sample.

## RESULTS

### Model selection

Table 2 displays the fit indices across models and illustrates that the AIC, BIC and aBIC values declined with the estimation of each additional class. That said, greater improvements in model fit occurred after adding the second and third classes, with a noticeable elbow bend in model fit improvements thereafter. Additionally, the models with five and six classes contained a first‐order derivative product matrix that was non‐positive definite. Comparisons of the two‐ and three‐class solutions revealed negligible differences in classification precision (Δentropy = −0.004) but that the three‐class solution identified an additional theoretically meaningful class. Further supporting the three‐class model, Table 3 displays the average probability that a participant belonged to a given class in the three‐class solution, revealing a high average likelihood that participants were correctly assigned to their given class (.84–.96) with only a small likelihood of misclassification.

**TABLE 2:** Model fit comparison of 1‐ to 6‐class latent class growth models.

Model	AIC	BIC	aBIC	ΔAIC	ΔBIC	ΔaBIC	Entropy	Class prevalence (%)					
								1	2	3	4	5	6
1 class	527,554.40	527,641.61	527,606.65	–	–	–	–	100	–	–	–	–	–
2 classes	517,321.97	517,464.66	517,407.46	10,232.44	10,176.94	10,199.19	0.864	85.72	14.28	–	–	–	–
**3 classes**	**511,732.63**	**511,930.82**	**511,851.37**	**5589.34**	**5533.85**	**5556.09**	**0.860**	**8.76**	**82.75**	**8.49**	–	–	–
4 classes	509,364.25	509,617.93	509,516.23	2368.38	2312.89	2335.13	0.846	9.34	7.72	77.75	5.20	–	–
5 classes^a^	506,207.49	506,516.66	506,392.72	3156.76	3101.26	3123.51	0.880	4.96	78.75	6.42	3.72	6.14	–
6 classes^a^	504,078.43	504,443.10	504,296.91	2129.06	2073.57	2095.81	0.866	6.30	4.62	5.08	75.71	4.64	3.65

*Note*: Selected solution presented in bold.

Abbreviations: aBIC, sample size adjusted Bayesian information criterion; AIC, Akaike information criterion; BIC, Bayesian information criterion.

^a^ Non‐positive definite first‐order derivative product matrix.

**TABLE 3:** Average latent class probabilities for most likely class membership (row) by latent class (column).

Class	*N*	%	1	2	3
Radicalized	1794	8.76%	**0.839**	0.097	0.064
Enfranchised	16,953	82.75%	0.032	**0.961**	0.007
Disenfranchised	1739	8.49%	0.081	0.057	**0.862**

*Note*: Values highlighted in bold reflect the average probability that a person estimated to belong to a given latent class was correctly categorized.

As a final test of the validity of the three‐class solution, we randomly split the sample into two halves and compared models with 1–6 class solutions across the two samples. These analyses produced solutions analogous to those found in the full sample (see Tables S1 and S2 in the Online Supplementary Materials). Thus, we opted for the three‐class solution as the best‐fitting model for our data.

### Class trajectories

**FIGURE 2:**
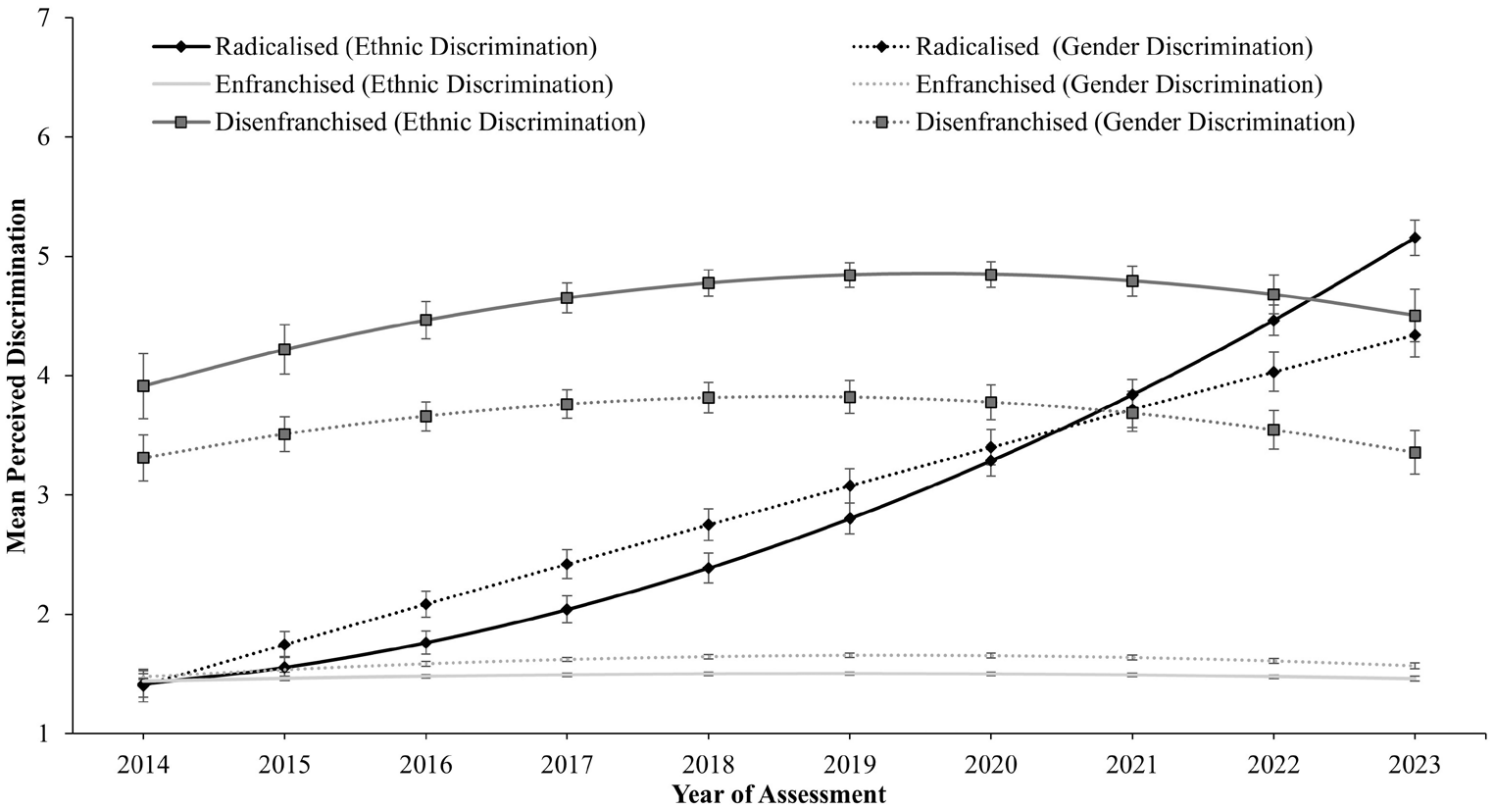
Three‐class latent class growth model of perceived ethnic and gender discrimination from 2014 to 2023. Error bars represent 95% confidence intervals.

Table 4 displays the parameter estimates for each latent class in the three‐class solution. As shown in Table 4, most of the sample (82.75%) displayed low mean levels of perceived ethnic and gender discrimination across assessment occasions (see also Figure 2), suggesting that most White men did not perceive themselves to be discriminated against based on their ethnicity or gender over the nine‐year period. We thus labelled this the *Enfranchised* class. Notably, Table 4 shows that the *Enfranchised* class displayed small linear increases in both perceived ethnic and gender discrimination, with a curvilinear decline over time.

**TABLE 4:** Parameter estimates for the three‐class latent growth model of perceived ethnic and gender discrimination.

Class	Perceived ethnic discrimination							Perceived gender discrimination						
	95% CI							95% CI						
		Estimate	*SE*	LB	UB	*p*‐value	Variance		Estimate	*SE*	LB	UB	*p*‐value	Variance
Enfranchised	*i*	1.50	0.01	1.484	1.515	<.001	0.26***	*i*	1.64	0.01	1.626	1.663	<.001	0.61***
	*s*	0.05	0.02	0.014	0.081	.006	0.00	*s*	0.17	0.02	0.128	0.204	<.001	0.00
	*q*	−0.26	0.05	−0.349	−0.165	<.001	0.00	*q*	−0.63	0.05	−0.735	−0.532	<.001	0.00
Disenfranchised	*i*	4.78	0.06	4.667	4.884	<.001	0.26***	*i*	3.82	0.07	3.687	3.943	<.001	0.61***
	*s*	0.96	0.20	0.566	1.351	<.001	0.00	*s*	0.29	0.15	0.003	0.583	.048	0.00
	*q*	−3.00	0.44	−3.869	−2.138	<.001	0.00	*q*	−2.42	0.36	−3.117	−1.715	<.001	0.00
Radicalized	*i*	2.39	0.06	2.262	2.511	<.001	0.26***	*i*	2.75	0.07	2.616	2.884	<.001	0.61***
	*s*	3.81	0.11	3.585	4.031	<.001	0.00	*s*	3.29	0.13	3.023	3.547	<.001	0.00
	*q*	3.46	0.35	2.774	4.140	<.001	0.00	*q*	−0.21	0.34	−0.878	0.466	.548	0.00

*Note*: Variances constrained to equality across latent classes.

Abbreviations: 95% CI, confidence interval; LB, lower bound; *i*, intercept; *q*, quadratic slope; *s*, linear slope; UB, upper bound.

*** *p* < .001.

A further 8.49% of the sample reported higher perceptions of ethnic and gender discrimination over time. This subgroup displayed linear increases in perceived ethnic and gender discrimination, but with a pronounced curvilinear decline over time. Additionally, Figure 1 illustrates that this subgroup scored moderately on perceived ethnic and gender discrimination over time. We therefore coined this subgroup the *Disenfranchised* class.

Finally, the remainder of the sample (8.76%) initially reported low levels of both ethnic and gender discrimination akin to the *Enfranchised* class (i.e. the 95% confidence intervals overlap; see Figure 1). However, this subgroup displayed considerable linear and curvilinear increases in perceived ethnic discrimination, as well as linear increases in perceived gender discrimination (see Table 4). Notably, this subgroup surpassed the *Disenfranchised* class in perceptions of both ethnic and gender discrimination at the final assessment occasion (2023; see Figure 2). Given these dramatic changes over time, we labelled this subgroup the *Radicalized* class.

### Predictors of class membership

To validate the distinctions between our three classes, we conducted a three‐step multi‐nominal logistic regression predicting class membership as a function of our socio‐demographic, identity and ideological covariates simultaneously.^3^ The three‐step approach first estimates the latent classes before assigning participants to the latent class to which they are most likely to belong. A multi‐nominal logistic regression is then performed to examine the predictors of the identified classes (after accounting for classification errors) without the predictors affecting the estimation of the latent classes themselves (Asparouhov & Muthén, 2014). Table 5 presents the results of these analyses using the largest class (the *Enfranchised*) as the basis for comparisons.

**TABLE 5:** Multi‐nomial logistic regression predicting the likelihood of belonging to the Disenfranchised and Radicalized classes (compared to the Enfranchised class).

Predictor	Disenfranchised (versus enfranchised)								Radicalized (versus enfranchised)							
	*B*	*SE*	95% CI		OR	95% CI		*p*	*B*	*SE*	95% CI		OR	95% CI		*p*
			LB	UB		LB	UB				LB	UB		LB	UB	
First assessment^a^	−0.10	0.16	−0.410	0.204	0.90	0.663	1.227	.512	−0.18	0.13	−0.433	0.071	0.83	0.649	1.073	.159
Born in NZ^b^	−0.38	0.09	−0.566	−0.202	0.68	0.568	0.817	<.001	0.13	0.09	−0.037	0.301	1.14	0.964	1.352	.124
Age (Time 6)	−0.66	0.24	−1.137	−0.183	0.52	0.321	0.833	.007	−0.88	0.21	−1.281	−0.474	0.42	0.278	0.622	<.001
IRD	1.32	0.18	0.971	1.659	3.73	2.640	5.255	<.001	0.67	0.14	0.388	0.942	1.95	1.475	2.566	<.001
GRD	2.48	0.20	2.088	2.863	11.89	8.068	17.513	<.001	1.47	0.18	1.118	1.827	4.36	3.058	6.217	<.001
Education	−0.22	0.16	−0.522	0.088	0.81	0.593	1.092	.163	0.18	0.13	−0.075	0.431	1.20	0.927	1.539	.169
Home ownership^b^	0.11	0.11	−0.114	0.324	1.11	0.892	1.382	.347	0.47	0.10	0.270	0.674	1.60	1.310	1.963	<.001
Sexual minority^b^	−0.26	0.15	−0.550	0.032	0.77	0.577	1.033	.081	0.16	0.11	−0.062	0.384	1.18	0.940	1.468	.156
Ethnic identity centrality	2.60	0.19	2.229	2.976	13.50	9.294	19.611	<.001	0.65	0.15	0.346	0.949	1.91	1.413	2.582	<.001
Gender identity centrality	−0.62	0.16	−0.940	−0.301	0.54	0.390	0.740	<.001	−0.04	0.13	−0.301	0.215	0.96	0.740	1.240	.743
Conservatism	1.51	0.22	1.078	1.934	4.51	2.938	6.918	<.001	1.22	0.18	0.872	1.561	3.38	2.391	4.764	<.001
System justification	−1.94	0.24	−2.409	−1.467	0.14	0.090	0.231	<.001	−0.49	0.21	−0.890	−0.088	0.61	0.410	0.916	.017
SDO	2.86	0.25	2.373	3.347	17.46	10.734	28.408	<.001	2.25	0.22	1.830	2.674	9.50	6.232	14.492	<.001
RWA	−0.36	0.25	−0.841	0.129	0.70	0.431	1.138	.150	−0.03	0.21	−0.435	0.372	0.97	0.647	1.450	.877
Conspiracy mentality	1.33	0.19	0.967	1.691	3.78	2.630	5.426	<.001	1.06	0.13	0.797	1.321	2.88	2.219	3.747	<.001

*Note*: Estimates are unstandardized, but all measures were rescaled on a common 0 to 1 metric.

Abbreviations: 95% CI, confidence interval; LB, lower bound; OR, odds ratio; UB, upper bound.

^a^ Participants' first completed assessment.

^b^ Dummy‐coded (0 = no, 1 = yes).

#### Disenfranchised versus Enfranchised

Table 5 shows that the odds of belonging to the *Disenfranchised* (versus *Enfranchised*) class were *lower* for those born in New Zealand, older (versus younger) people and people higher (versus lower) on gender identity centrality and system justification. Conversely, the odds of belonging to the *Disenfranchised* (versus *Enfranchised*) class were *higher* for people scoring high on IRD, GRD, ethnic identity centrality, conservatism, SDO and conspiracy mentality. The odds of belonging to these two classes did not, however, reliably vary by education, home ownership, sexual minority status, levels of RWA or by participants' first completed assessment.

#### Radicalized versus Enfranchised

Table 5 reveals that the odds of belonging to the *Radicalized* (vs. *Enfranchised*) class were lower for older (versus younger) people and those scoring high (vs. low) on system justification. However, the odds of belonging to the *Radicalized* (vs. *Enfranchised*) class were *higher* for homeowners, as well as those scoring high (versus low) on IRD, GRD, ethnic identity centrality, conservatism, SDO and conspiracy mentality. The odds of belonging to these two classes did not reliably vary by education, sexual minority status, gender identity centrality, RWA, whether participants were born in New Zealand or by participants' first completed assessment.

#### Radicalized versus Disenfranchised

To illustrate the distinctions between our two classes of interest, Table 6 displays the predictors of belonging to the *Radicalized* (vs. the *Disenfranchised*) class. As shown in Table 6, the odds of belonging to the *Radicalized* (vs. the *Disenfranchised*) class were lower for people scoring high on IRD, GRD and ethnic identity centrality. Conversely, the odds of belonging to the *Radicalized* (versus the *Disenfranchised*) class were *higher* for people born in New Zealand and people scoring high on gender identity centrality and system justification. Although home ownership, education and sexual minority status also increased the odds of belonging to the *Radicalized* (versus the *Disenfranchised*) class, these effects were relatively small. The odds of belonging to these two classes did not, however, differ by age, conservatism, SDO, RWA, conspiracy beliefs or participants' first completed assessment.

**TABLE 6:** Multi‐nomial logistic regression predicting the likelihood of belonging to the Radicalized class (compared to the Disenfranchised class).

Predictor	Radicalized (versus disenfranchised)							
	*B*	*SE*	95% CI		OR	95% CI		*p*
			LB	UB		LB	UB	
First assessment^a^	−0.08	0.20	−0.461	0.305	0.93	0.63	1.357	.690
Born in NZ^b^	0.52	0.12	0.274	0.759	1.68	1.315	2.136	<.001
Age (Time 6)	−0.22	0.30	−0.808	0.371	0.80	0.446	1.450	.468
IRD	−0.65	0.22	−1.079	−0.220	0.52	0.340	0.802	.003
GRD	−1.00	0.25	−1.492	−0.514	0.37	0.225	0.598	<.001
Education	0.40	0.20	0.008	0.782	1.48	1.008	2.186	.045
Home ownership^b^	0.37	0.15	0.073	0.662	1.44	1.075	1.939	.015
Sexual minority^b^	0.42	0.18	0.059	0.782	1.52	1.061	2.185	.023
Ethnic identity centrality	−1.96	0.24	−2.428	−1.483	0.14	0.088	0.227	<.001
Gender identity centrality	0.58	0.21	0.169	0.986	1.78	1.184	2.680	.006
Conservatism	−0.29	0.28	−0.832	0.253	0.75	0.435	1.287	.295
System justification	1.45	0.31	0.842	2.056	4.26	2.321	7.814	<.001
SDO	−0.61	0.31	−1.225	0.008	0.54	0.294	1.008	.053
RWA	0.32	0.31	−0.285	0.933	1.38	0.752	2.542	.297
Conspiracy mentality	−0.27	0.23	−0.723	0.183	0.76	0.485	1.201	.243

*Note*: Estimates are unstandardized, but all measures were rescaled on a common 0 to 1 metric.

Abbreviations: 95% CI, confidence interval; LB, lower bound; OR, odds ratio; UB, upper bound.

^a^ Participants' first completed assessment.

^b^ Dummy‐coded (0 = no, 1 = yes).

### Sociopolitical and well‐being differences

Finally, we examined whether the three classes differed in their socio‐political attitudes and subjective health and well‐being. To do so, we used a *distal* three‐step approach where class membership is used to predict our focal measures. We then used equality tests of the means to evaluate whether the three classes significantly differed from each other across measures (see Asparouhov & Muthén, 2014). Table S3 displays the means for each class and the overall equality tests of the means across our focal constructs.

#### Ideology and policy support

**FIGURE 3:**
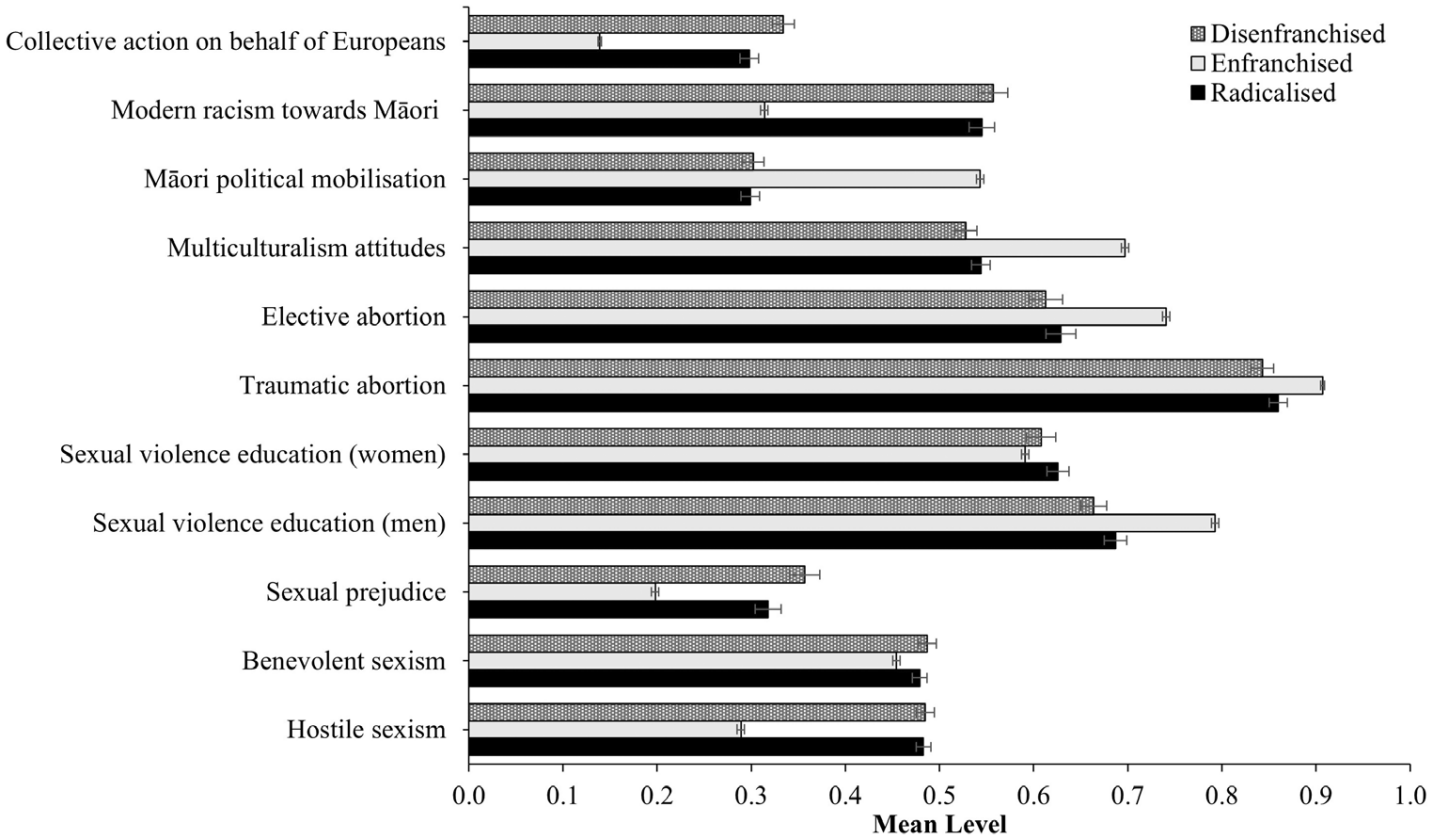
Mean ideology and policy support across classes. Constructs were rescaled on a 0 to 1 scale to facilitate comparisons. Error bars represent 95% confidence intervals.

Turning first to ideology and policy support, Figure 3 reveals that the *Enfranchised* class reported the lowest mean levels of hostile and benevolent sexism, sexual prejudice, support for sexual violence education targeting women, modern racism towards Māori and support for collective action on behalf of Europeans. Additionally, the *Enfranchised* class reported the highest mean levels of support for sexual violence prevention targeting men, traumatic abortion, elective abortion, multi‐culturalism and Māori political mobilization.

In contrast, the *Disenfranchised* class reported the highest mean levels of sexual prejudice and support for collective action on behalf of Europeans, as well as the lowest mean levels of support for traumatic abortion and sexual violence education targeting men. Finally, the *Radicalized* and *Disenfranchised* classes reported similar levels of hostile (*χ* ^2^_(1)_ = 0.14, *p* = .714) and benevolent (*χ* ^2^_(1)_ = 1.64, *p* = .201) sexism, support for sexual violence education for women (*χ* ^2^_(1)_ = 3.40, *p* = .065), elective abortion (*χ* ^2^_(1)_ = 1.87, *p* = .172), multi‐culturalism (*χ* ^2^_(1)_ = 3.58, *p* = .058), Māori political mobilization (*χ* ^2^_(1)_ = 0.17, *p* = .734) and modern racism towards Māori (*χ* ^2^_(1)_ = 1.32, *p* = .252). Taken together, these findings reveal that (a) the *Enfranchised* class reported the lowest levels of prejudice and the highest support for progressive policy and action, (b) the *Disenfranchised* reported the highest sexual prejudice and European collective action, but that (c) the *Disenfranchised* and *Radicalized* classes generally reported similar levels of sexism and opposition towards progressive policy and action.

#### Warmth towards ethnic and migrant groups

**FIGURE 4:**
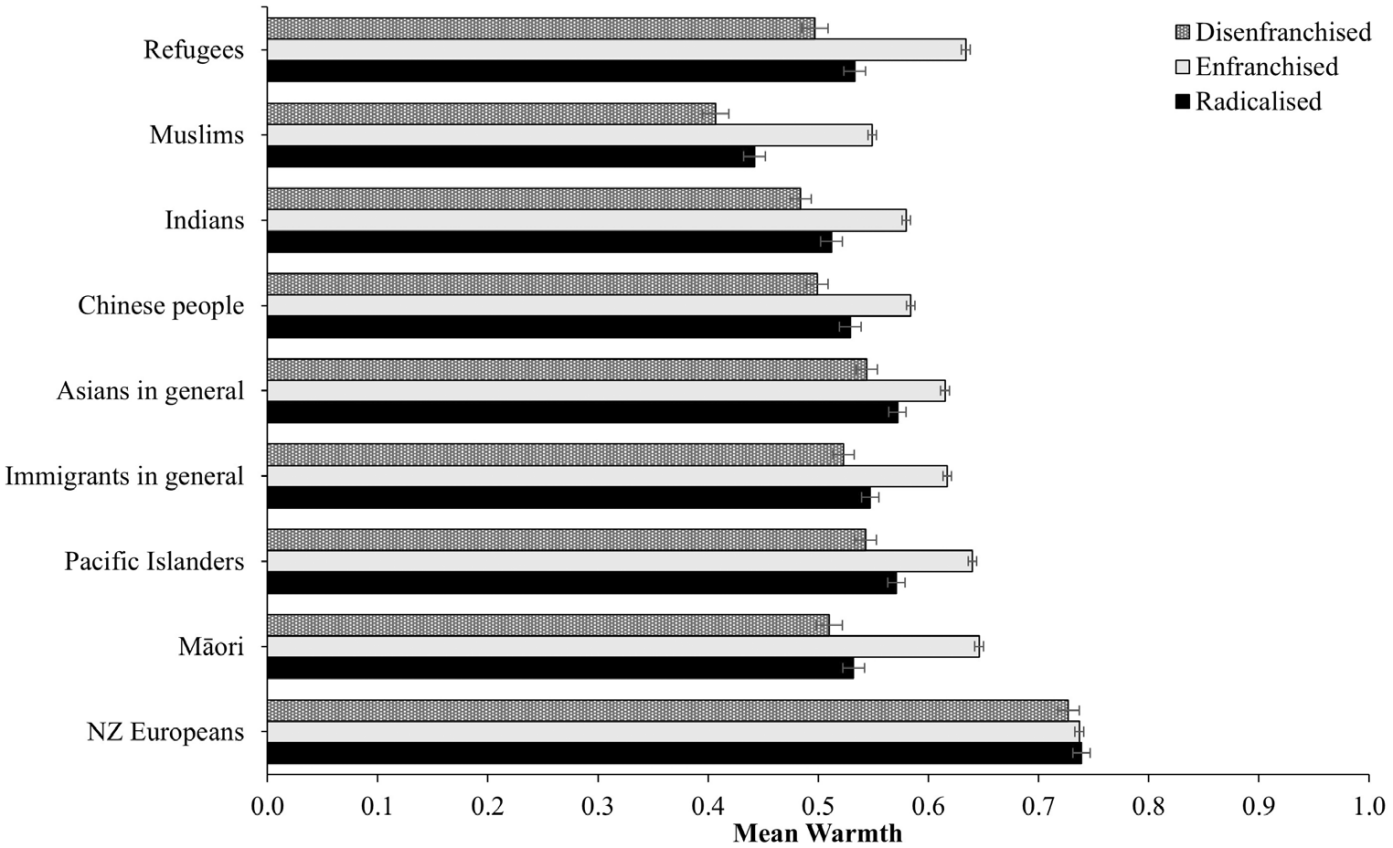
Mean feeling thermometer ratings across classes. Constructs were rescaled on a 0 to 1 scale to facilitate comparisons. Error bars represent 95% confidence intervals.

Figure 4 reveals consistent differences in feeling thermometer ratings across the three classes (see also Table S3). Namely, the *Enfranchised* class consistently reported higher mean levels of warmth towards ethnic minorities, immigrants and refugees. In contrast, the *Disenfranchised* class consistently reported the lowest warmth ratings towards these groups. Interestingly, no notable differences in warmth towards New Zealand Europeans emerged across classes (*χ* ^2^_(1)_ = 3.96, *p* = .138). Thus, differences across classes primarily emerged in attitudes towards *outgroups* rather than towards the European ingroup.

#### Political party support

**FIGURE 5:**
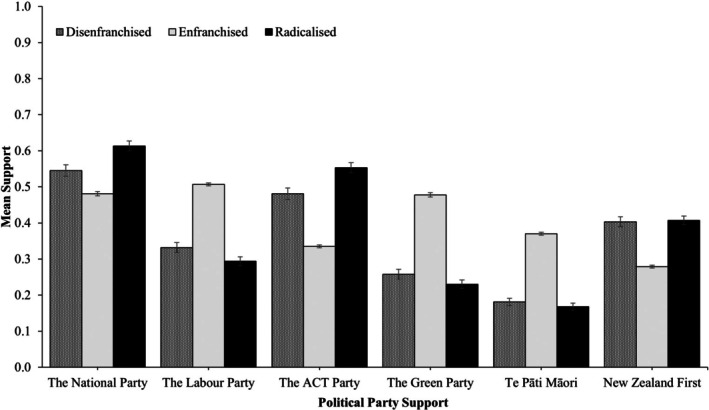
Political party support across classes. Constructs were rescaled on a 0 to 1 scale to facilitate comparisons. Error bars represent 95% confidence intervals.

Turning to political party support, differences emerged across classes for each of the major political parties in New Zealand (see Table S3 and Figure 5). First, support for the National party and the ACT party was highest among the *Radicalized*, followed by the *Disenfranchised* and, finally, the *Enfranchised* class. The *Disenfranchised* and *Radicalized* classes expressed comparable support for New Zealand First (*χ* ^2^_(1)_ = 0.25, *p* = .615) but were both higher than the *Enfranchised* class.

In contrast, support for the Labour Party and the Green Party was highest among the *Enfranchised*, followed by the *Disenfranchised* and the *Radicalized* class. Support for Te Pāti Māori (the Māori Party) was highest among the *Enfranchised* and similarly low among the *Disenfranchised* and *Radicalized* classes (*χ* ^2^_(1)_ = 3.31, *p* = .069).

#### Health and well‐being

**FIGURE 6:**
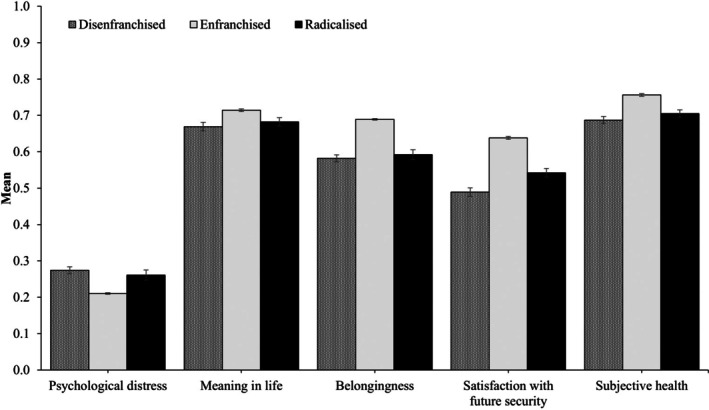
Mean subjective health and well‐being across classes. Constructs were rescaled on a 0 to 1 scale to facilitate comparisons. Error bars represent 95% confidence intervals.

Finally, we examined well‐being differences across our three profiles. As shown in Figure 6, the *Enfranchised* reported the lowest levels of psychological distress and the highest levels of subjective well‐being across measures. Conversely, the *Disenfranchised* reported the highest mean levels of psychological distress, as well as the lowest mean levels of financial security and subjective health satisfaction (see also Table S3). The *Radicalized* were similar to the *Disenfranchised* in meaning in life (*χ* ^2^_(1)_ = 2.69, *p* = .101) and felt belongingness (*χ* ^2^_(1)_ = 2.76, *p* = .097), although both scored lower on these constructs than the *Enfranchised* class.

## DISCUSSION

Although right‐wing political movements have stoked fears over claims of reverse discrimination (Simi et al., 2024), few studies examine the prevalence of perceived reverse discrimination, nor how these perceptions may change over time. The present study addressed these oversights via an exploratory analysis of perceived ethnic‐ and gender‐based discrimination in a longitudinal nationwide sample of White men. Reassuringly, our results suggest that most White men do not feel discriminated against—82.75% of our sample reported low and stable perceptions of both ethnic and gender‐based discrimination over time. That these perceptions are consistent with White men's structurally advantaged societal position alleviates concerns of widespread racial and gender‐based discontent among advantaged groups in New Zealand (e.g. Craymer, 2023).

Despite these encouraging results, our analyses also identified two subgroups of White men who *did* report feeling discriminated against based on their ethnicity and gender. First, we identified a *Disenfranchised* class (8.49% of the sample) who consistently reported moderate levels of perceived ethnic discrimination and low to moderate levels of gender discrimination over time. The *Disenfranchised* class was more likely to be young, born overseas and reported higher feelings of relative deprivation, poorer well‐being and lower satisfaction with their future security than the rest of the sample. Together, these results support prior work suggesting that White men with fewer material and structural resources may explain their socioeconomic disadvantage via racial discourses (e.g. Okuyan et al., 2023).

Additionally, the *Disenfranchised* class scored higher on conservatism and SDO, suggesting that perceived discrimination may be motivated by a desire for group‐based dominance and hierarchy among advantaged groups (see Bahamondes et al., 2021b). However, White men *lower* on system justification were also most likely to belong to the *Disenfranchised* class. Although this latter finding may be surprising initially, new research shows that reactionary movements are motivated by the perception that existing social systems are failing them and are in need of change (Caricati & Rossi, 2024; Liekefett & Becker, 2022; Lilly et al., 2025). Thus, despite privileging advantaged groups, White men may still *believe* that the status quo unfairly disadvantages them. In line with this possibility, the *Disenfranchised* class was also higher in conspiracy belief mentality, suggesting distrust of (or opposition to) mainstream social narratives (see also Thomas et al., 2024). Taken together, these results provide nuanced insights into a subgroup of White men who experience some forms of disadvantage but (inaccurately) attribute this disadvantage to their gender and ethnicity.

Concerningly, we also identified a subgroup of White men (8.76%) who initially reported similar levels of perceived discrimination to the *Enfranchised* class but substantially *increased* their perceptions of discrimination from 2016 onwards. The timing of this change aligns with the increasing prominence of alt‐right narratives following President Trump's first inauguration (2016), as well as increased discussions of gender and racial inequalities in the wake of the #MeToo (2017) and #BlackLivesMatter (2020) movements. This changing landscape may have spurred perceptions of discrimination among a group that previously felt content with their societal position. Our demographic and socio‐political correlates support this interpretation; the *Radicalized* class was more likely to be younger homeowners compared to the rest of the sample. Moreover, although the *Radicalized* were similar to the *Disenfranchised* class across ideological and well‐being measures, they were more likely to be born in New Zealand, reported lower levels of relative deprivation and reported higher system justification and support for right‐wing political parties (i.e. National and ACT).

Taken together, these results suggest that we identified two distinct ‘types’ of White men who feel discriminated against: (a) an economically disadvantaged group seeking to explain their disadvantage (the *Disenfranchised*) and (b) a more privileged group threatened by recent social progress for structurally disadvantaged groups (the *Radicalized*). Understanding these distinctions is integral to designing effective interventions to *mitigate* intergroup unrest and support for alt‐right movements among advantaged groups. For instance, tackling right‐wing media and conspiracies should reduce perceptions of threat and discrimination among White men more broadly, whereas perceived discrimination among the *Disenfranchised* may decline if government policy addresses the underlying causes of their disadvantage (i.e. economic precarity). However, the characteristics of the *Radicalized* class suggest that, despite their socioeconomic privilege, the *Radicalized* class feels threatened by recent societal changes and anticipates *future* status loss (Anier et al., 2016; Jetten et al., 2015). Thus, the *Radicalized* may see attempts to redress inequality as threats to their ‘deserved’ status (Schmitt & Branscombe, 2002) and communicate this status threat by claiming that others are the ‘true’ beneficiaries of favourable treatment (Billig, 1991; Wetherell & Potter, 1992). Such narratives are difficult to tackle because socioeconomically advantaged group members have more socio‐political power (although see Cabrera & Corces‐Zimmerman, 2019; Liu, 2017) and the desire to maintain a privileged position often elicits deleterious action (Liu, 2017; Simi et al., 2024). Thus, redressing both current economic precarity among the *Disenfranchised* and status anxiety among the *Radicalized* is required to deescalate perceptions of reverse discrimination and, ultimately, support for right‐wing reactionary politics and social movements. Although such interventions are beyond the scope of the present study, we encourage future research and policymakers to explore these possibilities.

### Caveats and future directions

Although the present study has several strengths, including the use of a robust, person‐centred methodology and large‐scale national data, there are limitations worthy of discussion. First, we used single‐item measures of perceived discrimination that, while useful in an omnibus survey design, prevent a more thorough interrogation of perceived discrimination among advantaged groups. For instance, we could not determine whether White men feel discriminated against in certain life domains (e.g. employment or education) or whether they felt discriminated against by certain groups (e.g. ethnic minorities or women). Doing so could identify *how* White men feel discriminated against and whether there are certain domains that elicit stronger opposition to minority groups and progressive social change. Moreover, our focus on annual assessments allows us to track perceptions of discrimination over a longer time period but prevents an examination of day‐to‐day encounters with (perceived) discrimination. Studies using shorter time intervals or experiential sampling methods would provide more nuanced insights into how and when advantaged groups feel discriminated against. Though beyond the scope of our data, the present study provides a springboard for future work testing these possibilities.

Additionally, although our short‐form scales were validated against their full‐form counterparts (Sibley et al., 2024), some measures (particularly relative deprivation and subjective health) displayed low internal consistency. Concerning relative deprivation, the low internal consistency is likely due to the items capturing distinct components of relative deprivation (that is, our relative deprivation measures both include a cognitive‐based measure of relative status and an affective measure of frustration at this status; Smith et al., 2012). Nonetheless, future work should use multi‐item scales to improve the reliability of these measures. Similarly, our reliance on 1‐ and 2‐item measures prevented tests of measurement invariance over time and across classes; future work should conduct such tests to account for measurement error.

We also caution against interpreting our results as revealing a universally ‘true’ number of classes or that certain demographics intrinsically predict class membership. LCGA allows us to identify participants' *most likely* class membership based on key socio‐demographic, identity and ideological constructs, but this approach cannot fully elucidate the causal processes underlying trajectories of change in perceived discrimination. Our approach towards missing data may also impact the differences identified between classes, as some participants did not complete our earliest assessments. Nonetheless, our study provides an ecologically valid, large‐scale examination of perceived discrimination in a general population sample and provides a critical foundation for future (quasi)experimental research exploring the causal processes underlying perceived discrimination among White men (see Diener et al., 2022, for discussion).

Finally, our results may vary across socio‐political contexts. New Zealand is highly egalitarian compared to other Western nations (see OECD, 2023) and is one of the most peaceful countries in the world (Global Peace Index, 2023). The classes identified in this study may differ in countries marked with greater interracial conflict or gender inequality. Likewise, subgroups of White men may be larger in countries with more established right‐wing movements utilizing status threat narratives (e.g. the United States; Berbrier, 2000). That said, the present study *did* identify two meaningful subgroups of White men who felt discriminated against and held more negative attitudes towards minorities and progressive social change. Moreover, anti‐White concerns played an important role in New Zealand's 2023 general election (Craymer, 2023), and the current right‐wing government continues to roll back Māori rights, including the abolishment of the Māori Health Authority and ongoing attempts to delegitimize Te Tiriti o Waitangi (the Treaty of Waitangi; Guenzler, 2024). That these patterns emerge in New Zealand speaks to the prevalence of status threat among advantaged groups globally and the need for cross‐national studies of perceived discrimination.

## CONCLUSION

Although contemporary politics is increasingly concerned about status threat among advantaged groups, few studies examine the extent to which advantaged groups perceive threats to their societal position. We addressed this oversight by examining perceived ethnic and gender discrimination among a nationwide longitudinal sample of White men. While most of our sample did not believe they were discriminated against based on ethnicity or gender, we identified two distinct subgroups who reported moderate levels of discrimination (the *Disenfranchised*) or reported initially low levels of discrimination that rapidly increased over time (the *Radicalized*). These two classes respectively captured subsets of materially disadvantaged and privileged White men who differed from the rest of the sample—and each other—across myriad socio‐political, identity and well‐being measures. The prevalence of these groups speaks to the need to monitor changes in perceived discrimination among advantaged groups to ensure that these groups do not become further radicalized over time.

## AUTHOR CONTRIBUTIONS

**Kieren J. Lilly:** Conceptualization; methodology; formal analysis (lead); writing – review and editing; writing – original draft; visualization. **Chantelle Kimberley:** Formal analysis; writing – review and editing. **Zoe Bertenshaw:** Writing – review and editing; formal analysis. **Joaquín Bahamondes:** Writing – review and editing. **Chris G. Sibley:** Conceptualization; methodology; data curation; funding acquisition; writing – review and editing. **Danny Osborne:** Conceptualization; methodology; writing – review and editing.

## CONFLICT OF INTEREST STATEMENT

The authors declare no conflicts of interest.

## Supporting information


**Table S1.** Model fit comparisons between solutions in random split halves of the sample.
**Table S2.** Parameter estimates for the three‐class latent growth model solutions using the random split halves of the sample.
**Table S3.** Mean sociopolitical attitudes and well‐being across classes.

## Data Availability

The data described in the paper are part of the New Zealand Attitudes and Values Study (NZAVS). Full copies of the NZAVS data files are held by all members of the NZAVS management team and advisory board. A de‐identified dataset containing the variables analysed in this manuscript is available upon request from the corresponding author, or any member of the NZAVS advisory board for the purposes of replication or checking of any published study using NZAVS data. The Mplus syntax used to test all models reported in this manuscript is available on the Open Science Framework: https://osf.io/75snb/.

## Footnotes

^1^
We did not conduct a priori power analyses, as our sample size was determined by our ability to retain participants. However, the minimum sample size recommendations for growth mixture modelling range from 200 to 4000, depending on the complexity of the model and missing response patterns (Kim, 2012). Our sample size considerably exceeds this requirement and allows for greater estimation accuracy.

^2^
Warmth towards refugees was measured from Times 9 through 15.

^3^
Given that we used each participant's earliest responses to our focal predictors, we also controlled for participants' first completed assessment (from Time 6 to Time 15).
