# Supplementary material for: Rise of the alt‐White? Examining the prevalence of perceived racial and gender discrimination among White men from 2014 to 2023
Source: Br J Soc Psychol. 2025 Aug 28;64(4):e70010. doi: 10.1111/bjso.70010 (PMC12392872; doi:10.1111/bjso.70010)
Supplement: Supplementary file 1 — Table S1. Model fit comparisons between solutions in random split halves of the sample. Table S2. Parameter estimates for the three‐class latent growth model solutions using the random split halves of the sample. Table S3. Mean sociopolitical attitudes and well‐being across classes. [file BJSO-64-0-s001.docx]

# Rise of the Alt-White? Examining the Prevalence of Perceived Racial and Gender Discrimination among White Men from 2014 to 2023

Online Supplementary Materials

**Table of Contents**

[Table S1. Model fit comparisons between solutions in random split halves of the sample. 2](#_Toc187388714)

[Table S2. Parameter estimates for the three-class latent growth model solutions using the random split halves of the sample. 3](#_Toc187388715)

[Table S3. Mean sociopolitical attitudes and well-being across classes. 4](#_Toc187388716)

## Table S1. *Model fit comparisons between solutions in random split halves of the sample.*

|  |  |  |  |  |  |  |  | **Class Prevalence (%)** | | | | | |
| --- | --- | --- | --- | --- | --- | --- | --- | --- | --- | --- | --- | --- | --- |
| **Model** | **AIC** | **BIC** | **aBIC** | **ΔAIC** | **ΔBIC** | **ΔaBIC** | **Entropy** | 1 | 2 | 3 | 4 | 5 | 6 |
| **Split half A** (*N* = 10,290) | | | | | | | | | | | | | |
| 1 class | 266732.18 | 266811.80 | 266776.85 | --- | --- | --- | 1.000 | 100.00 | --- | --- | --- | --- | --- |
| 2 classes | 261263.12 | 261393.42 | 261336.21 | 5469.06 | 5418.39 | 5440.63 | 0.866 | 14.56 | 85.44 | --- | --- | --- | --- |
| **3 classes** | **258483.06** | **258664.04** | **258584.59** | **2780.05** | **2729.38** | **2751.62** | **0.861** | **9.25** | **82.38** | **8.37** | --- | --- | --- |
| 4 classes | 257178.48 | 257410.13 | 257308.43 | 1304.58 | 1253.91 | 1276.16 | 0.857 | 6.06 | 80.93 | 6.93 | 6.07 | --- | --- |
| 5 classes | 255725.52 | 256007.84 | 255883.91 | 1452.96 | 1402.28 | 1424.53 | 0.869 | 77.21 | 7.25 | 4.68 | 6.79 | 4.06 | --- |
| 6 classes^⸷^ | 254615.52 | 254948.51 | 254802.33 | 1110.00 | 1059.33 | 1081.57 | 0.875 | 3.73 | 3.27 | 6.60 | 5.18 | 76.99 | 4.24 |
| **Split half B** (*N* = 10,196) | | | | | | | | | | | | | |
| 1 class | 260801.03 | 260880.56 | 260845.60 | --- | --- | --- | 1.000 | 100.00 | --- | --- | --- | --- | --- |
| 2 classes | 256047.76 | 256177.90 | 256120.70 | 4753.27 | 4702.66 | 4724.90 | 0.865 | 14.04 | 85.97 | --- | --- | --- | --- |
| **3 classes** | **253263.30** | **253444.04** | **253364.60** | **2784.47** | **2733.86** | **2756.10** | **0.860** | **8.46** | **8.44** | **83.10** | --- | --- | --- |
| 4 classes | 252031.80 | 252263.15 | 252161.46 | 1231.50 | 1180.89 | 1203.13 | 0.843 | 7.84 | 5.29 | 8.91 | 77.97 | --- | --- |
| 5 classes^⸷^ | 250402.15 | 250684.11 | 250560.17 | 1629.66 | 1579.05 | 1601.29 | 0.880 | 6.18 | 78.48 | 3.44 | 5.44 | 6.45 | --- |
| 6 classes^⸷^ | 249300.27 | 249632.83 | 249486.65 | 1101.88 | 1051.27 | 1073.52 | 0.859 | 75.64 | 5.54 | 3.70 | 5.92 | 5.20 | 4.00 |

*Note.* ^†^Non-positive definite first-order derivative product matrix. AIC = Akaike Information Criterion; BIC = Bayesian Information Criterion; aBIC = Sample-size adjusted Bayesian Information Criterion.

## Table S2. *Parameter estimates for the three-class latent growth model solutions using the random split halves of the sample.*

|  |  | **Perceived Ethnic Discrimination** | | | | | |  | **Perceived Gender Discrimination** | | | | | |
| --- | --- | --- | --- | --- | --- | --- | --- | --- | --- | --- | --- | --- | --- | --- |
|  |  |  |  | **95% CI** | |  |  |  |  |  | **95% CI** | |  |  |
| Class |  | Estimate | *SE* | LB | UB | *p*-value | Variance |  | Estimate | *SE* | LB | UB | *p*-value | Variance |
| **Split half A** (*N* = 10,290) | | | | | | | | | | | | | | |
| **Enfranchised** | *i* | 1.48 | 0.01 | 1.462 | 1.504 | < .001 | 0.26 |  | 1.63 | 0.01 | 1.604 | 1.655 | < .001 | 0.61 |
|  | *s* | 0.07 | 0.03 | 0.020 | 0.117 | .005 | 0.00 |  | 0.16 | 0.03 | 0.106 | 0.213 | < .001 | 0.00 |
|  | *q* | -0.21 | 0.07 | -0.345 | -0.083 | .001 | 0.00 |  | -0.55 | 0.07 | -0.696 | -0.406 | < .001 | 0.00 |
|  |  |  |  |  |  |  |  |  |  |  |  |  |  |  |
| **Disenfranchised** | *i* | 4.82 | 0.08 | 4.673 | 4.970 | < .001 | 0.26 |  | 3.88 | 0.09 | 3.696 | 4.066 | < .001 | 0.61 |
|  | *s* | 0.95 | 0.32 | 0.316 | 1.576 | .003 | 0.00 |  | 0.26 | 0.25 | -0.219 | 0.746 | .284 | 0.00 |
|  | *q* | -3.12 | 0.57 | -4.233 | -2.002 | < .001 | 0.00 |  | -2.20 | 0.53 | -3.243 | -1.161 | < .001 | 0.00 |
|  |  |  |  |  |  |  |  |  |  |  |  |  |  |  |
| **Radicalised** | *i* | 2.42 | 0.11 | 2.213 | 2.624 | < .001 | 0.26 |  | 2.78 | 0.11 | 2.571 | 2.990 | < .001 | 0.61 |
|  | *s* | 3.89 | 0.15 | 3.590 | 4.181 | < .001 | 0.00 |  | 3.35 | 0.19 | 2.967 | 3.725 | < .001 | 0.00 |
|  | *q* | 3.48 | 0.52 | 2.456 | 4.497 | < .001 | 0.00 |  | -0.39 | 0.50 | -1.370 | 0.598 | .442 | 0.00 |
|  |  |  |  |  |  |  |  |  |  |  |  |  |  |  |
| **Split half B** (*N* = 10,196) | | | | | | | | | | | | | | |
| **Enfranchised** | *i* | 1.52 | 0.01 | 1.494 | 1.538 | < .001 | 0.25 |  | 1.66 | 0.01 | 1.630 | 1.685 | < .001 | 0.60 |
|  | *s* | 0.03 | 0.02 | -0.021 | 0.074 | .273 | 0.00 |  | 0.17 | 0.03 | 0.115 | 0.225 | < .001 | 0.00 |
|  | *q* | -0.30 | 0.07 | -0.434 | -0.171 | .001 | 0.00 |  | -0.72 | 0.07 | -0.860 | -0.575 | < .001 | 0.00 |
|  |  |  |  |  |  |  |  |  |  |  |  |  |  |  |
| **Disenfranchised** | *i* | 4.72 | 0.08 | 4.559 | 4.880 | < .001 | 0.25 |  | 3.74 | 0.09 | 3.561 | 3.924 | < .001 | 0.60 |
|  | *s* | 0.96 | 0.26 | 0.446 | 1.465 | < .001 | 0.00 |  | 0.31 | 0.19 | -0.055 | 0.675 | .096 | 0.00 |
|  | *q* | -2.79 | 0.70 | -4.158 | -1.413 | < .001 | 0.00 |  | -2.57 | 0.51 | -3.561 | -1.574 | < .001 | 0.00 |
|  |  |  |  |  |  |  |  |  |  |  |  |  |  |  |
| **Radicalised** | *i* | 2.35 | 0.08 | 2.196 | 2.513 | < .001 | 0.25 |  | 2.72 | 0.09 | 2.546 | 2.903 | < .001 | 0.60 |
|  | *s* | 3.71 | 0.19 | 3.340 | 4.077 | < .001 | 0.00 |  | 3.23 | 0.19 | 2.859 | 3.596 | < .001 | 0.00 |
|  | *q* | 3.39 | 0.49 | 2.433 | 4.353 | < .001 | 0.00 |  | -0.01 | 0.48 | -0.954 | 0.943 | .990 | 0.00 |
|  |  |  |  |  |  |  |  |  |  |  |  |  |  |  |

## Table S3. *Mean sociopolitical attitudes and well-being across classes.*

|  | **Enfranchised** | | **Disenfranchised** | | **Radicalised** | | **Overall chi-square tests** | | |
| --- | --- | --- | --- | --- | --- | --- | --- | --- | --- |
| **Variable** | *Mean* | *SE* | *Mean* | *SE* | *Mean* | *SE* | χ2 | df | p-value |
| **Ideology and policy support** |  |  |  |  |  |  |  |  |  |
| Hostile sexism | 0.29 | 0.002 | 0.49 | 0.005 | 0.48 | 0.004 | 2759.90 | 2 | < .001 |
| Benevolent sexism | 0.45 | 0.002 | 0.49 | 0.005 | 0.48 | 0.004 | 66.28 | 2 | < .001 |
| Sexual prejudice | 0.20 | 0.002 | 0.36 | 0.008 | 0.32 | 0.007 | 562.88 | 2 | < .001 |
| Sexual violence education (men) | 0.79 | 0.002 | 0.66 | 0.007 | 0.69 | 0.006 | 520.20 | 2 | < .001 |
| Sexual violence education (women) | 0.59 | 0.002 | 0.61 | 0.008 | 0.63 | 0.006 | 27.56 | 2 | < .001 |
| Traumatic abortion | 0.91 | 0.001 | 0.84 | 0.006 | 0.86 | 0.005 | 166.82 | 2 | < .001 |
| Elective abortion | 0.74 | 0.002 | 0.61 | 0.009 | 0.63 | 0.008 | 358.84 | 2 | < .001 |
| Multiculturalism attitudes | 0.70 | 0.002 | 0.53 | 0.006 | 0.54 | 0.005 | 1267.01 | 2 | < .001 |
| Māori political mobilisation | 0.54 | 0.002 | 0.30 | 0.006 | 0.30 | 0.005 | 2799.46 | 2 | < .001 |
| Modern racism towards Māori | 0.31 | 0.002 | 0.56 | 0.008 | 0.55 | 0.007 | 1929.95 | 2 | < .001 |
| Collective action on behalf of Europeans | 0.14 | 0.001 | 0.33 | 0.006 | 0.30 | 0.005 | 1636.09 | 2 | < .001 |
| **Warmth Ratings** |  |  |  |  |  |  |  |  |  |
| NZ Europeans | 0.74 | 0.002 | 0.73 | 0.005 | 0.74 | 0.004 | 3.96 | 2 | .138 |
| Māori | 0.65 | 0.002 | 0.51 | 0.006 | 0.53 | 0.005 | 927.48 | 2 | < .001 |
| Pacific Islanders | 0.64 | 0.002 | 0.54 | 0.005 | 0.57 | 0.004 | 518.94 | 2 | < .001 |
| Immigrants in general | 0.62 | 0.002 | 0.52 | 0.005 | 0.55 | 0.004 | 514.57 | 2 | < .001 |
| Asians in general | 0.62 | 0.002 | 0.54 | 0.005 | 0.57 | 0.004 | 242.48 | 2 | < .001 |
| Chinese | 0.58 | 0.002 | 0.50 | 0.005 | 0.53 | 0.005 | 322.84 | 2 | < .001 |
| Indians | 0.58 | 0.002 | 0.48 | 0.005 | 0.51 | 0.005 | 411.95 | 2 | < .001 |
| Muslims | 0.55 | 0.002 | 0.41 | 0.006 | 0.44 | 0.005 | 807.58 | 2 | < .001 |
| Refugees | 0.63 | 0.002 | 0.50 | 0.006 | 0.53 | 0.005 | 792.27 | 2 | < .001 |
| **Political party support** |  |  |  |  |  |  |  |  |  |
| The National Party | 0.48 | 0.003 | 0.55 | 0.008 | 0.61 | 0.007 | 399.97 | 2 | < .001 |
| The Labour Party | 0.51 | 0.002 | 0.33 | 0.007 | 0.29 | 0.006 | 1436.99 | 2 | < .001 |
| The ACT Party | 0.34 | 0.002 | 0.48 | 0.008 | 0.55 | 0.007 | 1103.98 | 2 | < .001 |
| The Green Party | 0.48 | 0.003 | 0.26 | 0.007 | 0.23 | 0.006 | 2079.60 | 2 | < .001 |
| Te Pāti Māori/The Māori Party | 0.37 | 0.002 | 0.18 | 0.005 | 0.17 | 0.005 | 2209.36 | 2 | < .001 |
| New Zealand First | 0.28 | 0.002 | 0.40 | 0.007 | 0.41 | 0.006 | 639.84 | 2 | < .001 |
| **Health and Well-being**^a^ |  |  |  |  |  |  |  |  |  |
| Psychological distress | 0.21 | 0.001 | 0.27 | 0.005 | 0.26 | 0.004 | 287.90 | 2 | < .001 |
| Meaning in life | 0.71 | 0.002 | 0.67 | 0.006 | 0.68 | 0.005 | 89.33 | 2 | < .001 |
| Belongingness | 0.69 | 0.001 | 0.58 | 0.005 | 0.59 | 0.004 | 882.37 | 2 | < .001 |
| Satisfaction with future security | 0.64 | 0.002 | 0.49 | 0.006 | 0.54 | 0.006 | 743.16 | 2 | < .001 |
| Subjective health | 0.76 | 0.002 | 0.69 | 0.005 | 0.71 | 0.005 | 237.15 | 2 | < .001 |
|  |  |  |  |  |  |  |  |  |  |
